# Effectiveness of a Walking Program Involving the Hybrid Assistive Limb Robotic Exoskeleton Suit for Improving Walking Ability in Stroke Patients: Protocol for a Randomized Controlled Trial

**DOI:** 10.2196/14001

**Published:** 2019-10-11

**Authors:** Hideo Tsurushima, Masafumi Mizukami, Kenichi Yoshikawa, Tomoyuki Ueno, Yasushi Hada, Masahiko Gosho, Yutaka Kohno, Koichi Hashimoto, Yuichi Iizumi, Toshihiro Kikuchi, Akira Matsumura, Hit2016 Study Group

**Affiliations:** 1 Department of Neurosurgery, Faculty of Medicine University of Tsukuba Tsukuba Japan; 2 Department of Physical Therapy, Ibaraki Prefectural University of Health Sciences Hospital Ami Japan; 3 Department of of Biostatistics, Faculty of Medicine, University of Tsukuba Tsukuba Japan; 4 Tsukuba Clinical Research & Development Organization, University of Tsukuba Tsukuba Japan

**Keywords:** Hybrid Assistive Limb (HAL), gait training, stroke, hemiparesis

## Abstract

**Background:**

Gait disturbance often occurs in stroke survivors. Recovery of walking function is challenging, as some gait disturbance due to hemiparesis often remains even after rehabilitation therapy, presenting a major obstacle towards regaining activities-of-daily-living performance and achieving social reintegration.

**Objective:**

This study aims to clarify the effectiveness of a walking program involving the wearable Hybrid Assistive Limb (HAL-TS01) robotic exoskeleton for improving walking ability in stroke patients with hemiparesis and stagnant recovery despite ongoing rehabilitation.

**Methods:**

This is a multicenter, randomized, parallel-group, controlled study (HAL group, n=27; control group, n=27). The study period includes preintervention observation (until stagnant recovery), intervention (HAL-based walking therapy or conventional rehabilitation; 5 weeks), and postintervention observation (2 weeks). Following provision of informed consent and primary registration, the patients undergo conventional rehabilitation for preintervention observation, during which the recovery of walking ability is monitored to identify patients with stagnant recovery (based on weekly assessments using the 10-meter maximum walking speed [MWS] test). Patients with an MWS of 30-60 m/minute and insufficient weekly improvement in MWS undergo secondary registration and are randomly assigned to undergo HAL-based walking therapy (HAL group) or conventional rehabilitation (control group). The primary outcome is the change in MWS from baseline to the end of the 5-week intervention.

**Results:**

This study began in November 2016 and is being conducted at 15 participating facilities in Japan.

**Conclusions:**

Assessments of walking ability vary greatly and it is difficult to define the threshold for significant differences. To reduce such variability, our study involves conducting conventional rehabilitation to the point of saturation before starting the intervention. Stagnation in the recovery of walking ability despite conventional rehabilitation highlights the limits of current medical care. The present study may bring evidence that HAL-based therapy can overcome such limitations and induce added recovery of walking ability, which would promote the use of HAL technology in the clinical setting.

**Trial Registration:**

UMIN Clinical Trials Registry UMIN000024805; https://upload.umin.ac.jp/cgi-open-bin/ctr_e/ctr_view.cgi?recptno=R000028545

## Introduction

At stroke onset, more than 60% of patients have gait disturbance and about 50% are unable to walk [1,2]. Recovery of walking function is generally a long and arduous process [1,3,4]. Current rehabilitation strategies for poststroke gait disturbance are quite effective, but patients with severe gait disturbance may not fully recover their walking ability. Furthermore, such gait disturbance is a major obstacle towards recovering activities-of -daily-living (ADL) performance and achieving social reintegration.

The Hybrid Assistive Limb (HAL) manufactured by Cyberdyne Inc. (Tsukuba, Japan) is a robotic exoskeleton that aids body movements by detecting and enhancing bioelectric signals to the muscles. When a person tries to move a muscle, a signal is transmitted from the brain through the spinal cord and motor neurons to said muscle, causing the musculoskeletal system to move. This process is accompanied by weak bioelectric signals detectable at the surface of the skin. The HAL was developed to read such bioelectric signals and help with movement per the person’s intentions [5,6]. Different hypotheses have been proposed about the effects of HAL therapy, including the interactive bio-feedback hypothesis and neural plasticity [7], but these remain unproven. Clinical studies conducted in Japan confirmed the clinical effects of HAL therapy and, in November 2015, a leg-type bipedal HAL was approved as a new medical device for managing spinal muscular atrophy, spinal and bulbar muscular atrophy, amyotrophic lateral sclerosis, Charcot-Marie-Tooth disease, distal myopathy, inclusion body myositis, congenital myopathy, and muscular dystrophy, apart from this clinical trial.

Whereas the mechanisms underlying the therapeutic action of the HAL have not been completely clarified, some researchers believed that HAL therapy is superior to standard robot-assisted training because it supplies better feedback and supports natural motions. Several earlier studies have examined the effectiveness of HAL-based walking exercises for alleviating gait disturbance in stroke patients. In a study on the stages of recovery after stroke, Kawamoto et al [8] showed that 16 sessions of HAL-based walking exercise increased walking speed and improved balance ability. In a randomized pilot trial, Watanabe et al [9] also found that 12 sessions of HAL-based walking exercise were more effective than conventional walking rehabilitation in terms of improving the functional ambulation category. Thus, these papers have confirmed the feasibility of HAL for clinical application.

Unlike prior studies, the present trial is designed to examine the effectiveness of HAL-based walking exercise in stroke patients with stagnant recovery under conventional rehabilitation. This is necessary because there is considerable variability on how recovery is assessed and what constitutes a meaningful rate of improvement. Therefore, the present study includes a preintervention observation period during which conventional rehabilitation for walking ability is performed until stagnation of recovery is noted. The fact that some patients no longer experience an improvement in walking ability despite continuing rehabilitation therapy highlights the current limits of conventional rehabilitation programs. The present study may bring evidence that HAL-based intervention can induce added recovery of walking ability in such patients, which would promote the use of HAL-based walking therapy in the clinical setting.

## Methods

### Study Objectives

The present study aims to clarify the efficacy of a HAL-based walking program versus conventional rehabilitation focused on restoring walking ability in patients with hemiparesis due to stroke.

### Study Design

The present study is designed as a multicenter, randomized, parallel-group, controlled trial. Our aim is to enroll 54 participants (27 in the HAL group and 27 in the control group) by December 2019. The patients have been recruited since November 2016, and the intervention is being conducted at 15 participating facilities.

**Figure 1:**
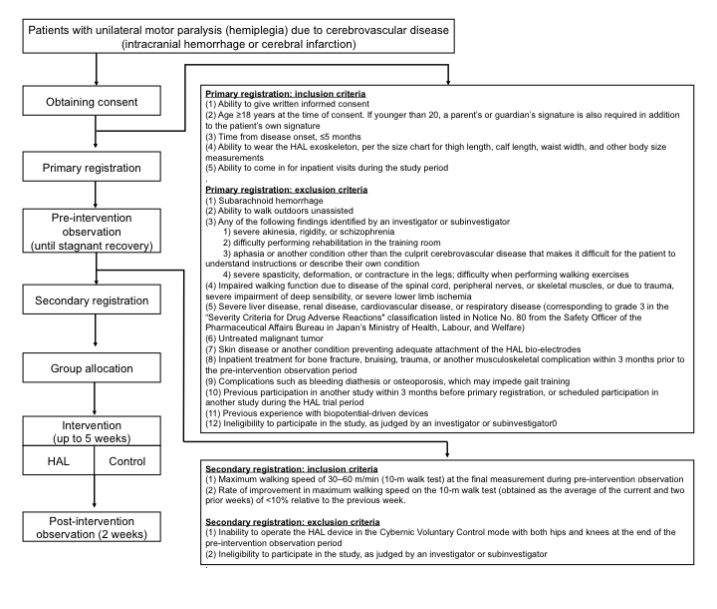
Study design. This multicenter, randomized, parallel-group, controlled study has three phases (preintervention observation, intervention, and postintervention observation). Following primary registration, the patients undergo conventional rehabilitation and are monitored to detect stagnant recovery. Upon secondary registration prior to the intervention, the patients are randomized to undergo conventional rehabilitation aimed at regaining walking ability (control group), or a walking program involving the use of the Hybrid Assistive Limb (HAL) robotic exoskeleton (HAL group).

The study has three phases (preintervention observation, intervention, and postintervention observation, as seen in Figure 1). Because of the evaluation of treatments that improve walking ability, this clinical trial focuses on walking ability rather than the severity of stroke to evaluate the patients.

**Figure 2:**
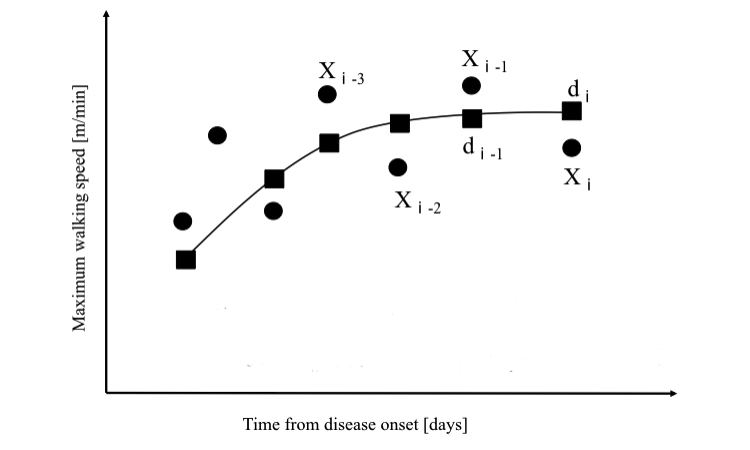
Method for smoothing the value of the maximum walking speed on the weekly 10-m walk test. MWS: maximum walking speed. (Xⅰ): MWS value measured for week i. (dⅰ): Smoothed MWS value for week i, obtained as a moving average of the MWS values measured for weeks i, i-1, and i-2.

The primary outcome of the study is the change in maximum walking speed (MWS) on the 10-m walk test from baseline to the end of the 5-week intervention. However, patients’ ADL or social reintegration are the true outcome, with walking ability selected as the surrogate outcome that was strongly associated with ADL or social reintegration [10]. In order to clarify whether the HAL-based walking program can boost walking ability once recovery has become stagnant despite ongoing conventional rehabilitation, the intervention is indicated only for patients who exhibit stagnant recovery of MWS, which is defined as insufficient weekly improvement in the MWS during the preintervention observation period. In addition to MWS, walking ability is also evaluated based on a patient’s ability to walk independently, but in this clinical trial the patients were only assessed with MWS because of its quantitative nature. The MWS values are measured every week and then smoothed over the current and previous two weeks (Standalone Equation 1) (Figure 2). The weekly rate of change in the smoothed MWS is obtained to determine the recovery rate (Standalone Equation 2). Stagnant recovery of MWS is then defined as <10% improvement compared to the previous week. Patients with an MWS of 30-60 m/minute and stagnant recovery of MWS are randomly assigned to either the control group or the HAL group.

This protocol has been approved by the Central Institutional Review Board of the Ibaraki Clinical Trial & Research Network. The research leader has notified the regulatory authorities with respect to the design and schedule of the clinical trial and has registered the study with the University Hospital Medical Information Network (UMIN trial registration number: UMIN000024805) prior to starting patient registration. Candidate patients are enrolled only upon supplying written informed consent for participation.

### Registration and Randomization

Upon supplying written informed consent, the patients were evaluated for eligibility according to the inclusion and exclusion criteria for primary registration. Registered patients who had completed the preintervention observation were evaluated for eligibility according to the inclusion and exclusion criteria for secondary registration. Stratified randomization was conducted, with MWS at allocation (<45 or ≥45 m/minute), age at the time of consent (<60 or ≥60 years), and the intervention center as the stratification factors. Data program and randomization program were served by CIMIC Co., Ltd. (Tokyo, Japan).

### Inclusion and Exclusion Criteria

Two sets of inclusion and exclusion criteria were employed in this study, namely at the time of primary and secondary registration. The inclusion criteria for primary registration included: (1) hemiparesis due to stroke; (2) age ≥18 years; (3) ≤5 months from stroke onset; and (4) ability to give written informed consent. Furthermore, patients had to be able to come in for inpatient evaluations during the study period and had to be able to wear and use the HAL robotic exoskeleton. Patients with subarachnoid hemorrhage, which involves bleeding in the subarachnoid space and is thus distinct from brain hemorrhage or cerebral ischemia, were excluded. Patients who achieved enough improvement in walking ability and become able to walk outdoors unassisted were also excluded, as they might not have benefitted as much from HAL therapy. For safety reasons and due to the specific nature of the device and training program, patients with any of the following conditions were also excluded: (1) severe akinesia, rigidity, or schizophrenia; (2) difficulty performing rehabilitation in the training room; (3) a condition other than the culprit cerebrovascular disease that makes it difficult for the patient to understand instructions; (4) impaired walking function due to a disease of the spinal cord, peripheral nerves, or skeletal muscles, or due to trauma, severe impairment of deep sensibility, or severe lower limb ischemia; or (5) other severe uncontrollable diseases.

The inclusion criteria for secondary registration were: (1) insufficient rate of weekly improvement in MWS (smoothed value) during preintervention observation (<10% improvement relative to the previous week); and (2) MWS of 30-60 m/minute at the final measurement during preintervention observation. Among patients who are able to wear the HAL robotic suit, we excluded those unable to operate it in the Cybernic Voluntary Control mode with both hips and knees, in which it can assist with movement in accordance with the person’s intentions.

### Discontinuation From the Study

Investigators and other persons involved are permitted to discontinue the study if consent is withdrawn, if it is discovered that an exclusion criteria is satisfied, if an adverse event or another circumstance renders the patient unable to participate in the intervention for ≥1 week at a time, or if it is deemed difficult to continue the study and valid to discontinue it for efficacy or safety reasons.

### Intervention

Following primary registration, the patients underwent conventional rehabilitation and were monitored to detect stagnant recovery (preintervention observation). Upon secondary registration prior to the intervention, the patients were randomized into two groups (intervention). Patients assigned to the control group underwent conventional rehabilitation aimed at restoring walking ability for 5 weeks, which did not involve the use of the HAL. Patients assigned to the HAL group underwent a rehabilitation program involving walking exercises performed while wearing the HAL robotic exoskeleton suit for 5 weeks. All patients underwent a 60-minute session of conventional rehabilitation followed by 20 minutes of rehabilitation over ground, which aimed to improve walking ability. This was conducted while wearing the HAL robotized suit (in the HAL group) or without wearing the HAL suit (in the control group), depending on group allocation. The HAL walking program took place for a net 20 minutes, except for the time involved with attaching and detaching the HAL. Following an intervention period, the patients continued conventional rehabilitation for another two weeks (postintervention observation). Postintervention observation is meant to evaluate whether the patient can maintain their improved walking ability, though it is short.

Conventional rehabilitation consists of 80-minute sessions of exercises designed to improve muscle tone in the paralyzed limbs, as well as muscle strength and coordination, thus enhancing basic motor skills, balance, and walking ability in preintervention observation and postintervention observation (Textbox 1). Conventional rehabilitation is conducted, in principle, 5 days per week.

**Conventional rehabilitation program.**

- Rehabilitation aimed at restoring walking ability:
  - Walking exercise (adaptive walking, outdoor walking, stair climbing/descending)
  - Treadmill walking exercise (with body weight support)
  - Endurance training (cycle ergometer exercise)
- Other rehabilitation therapy

### Assessment and Study Endpoints

The primary outcome is the change in MWS from baseline (ie, right before the intervention) to the end of the 5-week intervention. The secondary outcomes include: (1) the change in mean step length and the change in average step rate at MWS; (2) the change in single-leg support time expressed as a percentage of the gait cycle at MWS; (3) the change in maximum distance walked over the course of 6 minutes; (4) the change in functional ambulation category; (5) the change in Berg Balance Scale score; and (6) the change in leg score upon Fugl-Meyer Assessment. Multimedia Appendix 1 provides an overview of the assessment schedule.

Preintervention observation during conventional rehabilitation is conducted until stagnant recovery. Intervention with HAL-based walking therapy or conventional rehabilitation is conducted for up to 5 weeks.

Safety was assessed in terms of the nature and frequency of adverse events occurring between the start of the intervention until the end of the postintervention observation period. The circumstances and frequency of HAL malfunction are also recorded.

A physical therapist (PT) group operating HAL and a PT group evaluating patients were created at each facility, and these two groups did not share patient information. A four-hour briefing session on protocols and measurement methods was held at all centers to equalize the quality of measurement methods. Attendance of this briefing session was a requirement for the staff to take part in this clinical trial.

### Sample Size

The main purpose of this study is to confirm the superiority of the HAL-based walking exercise (HAL group) over conventional rehabilitation focused on improving walking ability (control group), and the main outcome is MWS improvement. Based on a previous clinical study [10] that compared a HAL group and a non-HAL group, and taking into consideration the fact that this is a multicenter study, the clinically meaningful between-group differences in mean MWS and associated standard deviation were conservatively estimated at 9 m/minute and 11 m/minute, respectively. A minimum sample size of 50 participants (25 patients per group) is expected to provide a power of 0.8 for detecting such differences, with two-sided significance of 0.05. Assuming a dropout rate of around 5%, we aim to include 54 participants (27 per group).

### Statistical Analysis

The efficacy of HAL-based therapy will be analyzed using the full set of data based on the intention-to-treat principle, and using the per-protocol set. The change in MWS from baseline (right before intervention) to week 5 of the intervention, which is the primary outcome, will be compared between the HAL group and the control group using a mixed model, repeated-measures analysis that includes group and intervention period (weeks 1-5) as factors, and group × period interaction, baseline MWS, and age as covariates. The change in mean step length and the change in average step rate at MWS will be similarly analyzed. Other secondary endpoints at 5 weeks will be analyzed using analysis of covariance, which will include group as a factor, and relevant baseline values and age as covariates. The number and percentage of patients who develop adverse events will be summarized for each group and compared between the two groups using Fisher’s exact test. The level of significance is set at 0.05 for all analyses.

## Results

This study began in November 2016 and is being conducted at 15 participating facilities in Japan. The study is in progress and the patient enrollment period is scheduled to end in December 2019.

## Discussion

This study is being conducted in Japan as a doctor-initiated clinical study to verify the effectiveness of robot-assisted therapy in the wider context of facilitating effective social reintegration of patients with stroke. Therefore, if the study can provide evidence of the superiority of the HAL-based walking exercise over conventional rehabilitation therapy, this would promote the use of HAL-based therapy in clinical practice.

The use of HAL-based exercise to restore motor ability after hemiparesis caused by stroke is likely to have the following medical and social effects: (1) improved motor ability due to recovery of central nervous function through HAL-assisted feedback of active, repeated motion; (2) reduced duration of hospitalization and rehabilitation; (3) fewer sequelae and reduced load on care givers; (4) amelioration of muscle weakness, muscle atrophy, and reduced joint range of motion caused by disuse, as well as better maintenance of ADL performance; and (5) compared to conventional assistance tools, HAL would provide more functionality to reduce residual sequelae such as impaired motor function in the limbs. To clarify these matters, it is necessary to design new study protocols that account for differences in underlying pathology, including symptoms and disease stage. It is also important to prove the effectiveness of the HAL-walking program in this clinical trial.

Rehabilitation is often provided for gait disturbance due to hemiparesis in patients with stroke. However, depending on the severity of such a disturbance, the patients may experience stagnation in their recovery of walking ability and fail to become capable of unassisted walking. A previous clinical study [11] reported that the HAL-based walking program helped increase walking ability in patients with stagnant recovery, suggesting that HAL-based walking exercise can overcome the limitations of conventional rehabilitation and boost walking ability even in patients with stagnant recovery. Considering that walking ability is a major factor in ADL function [10,12-15], benefits conferred by the HAL-based walking program would contribute greatly to enhancing ADL function.

In our present study, stagnant recovery of walking ability was assessed during a preintervention observation period, and only patients who showed insufficient weekly improvement continued to the intervention. This approach will allow us to determine whether the HAL-based walking program can boost walking ability in patients with stagnant recovery despite ongoing rehabilitation.

Because it is a simple and accessible indicator of motor ability during gait, MWS is the most commonly used indicator for assessing walking ability. MWS is also used as an indicator of therapeutic effect, which is helpful for formulating a treatment plan and predicting the prognosis of rehabilitation for impaired motor function in elderly patients or patients with stroke. The minimal difference in MWS that is clinically significant during recovery after stroke has been reported at 8.4 m/minute by Perera et al [16] and at 9.6 m/minute by Tilson et al [17]. While stagnant recovery was often described in studies assessing walking ability or ADL function in patients with stroke [1,2,18], no consensus has been reached on a method for defining the threshold for recovery stagnation. An earlier study defined recovery stagnation in terms of the weekly rate of improvement in MWS, leading to the formulation of guidelines. Moreover, MWS is a useful parameter in the context of social reintegration, which is facilitated through improvement of walking function in patients who are capable of unassisted walking in the home but not outside. Therefore, we set two MWS-based inclusion criteria for the secondary registration. Prior research suggests that the cut-off value for walking speed allowing restricted community walking is 0.4 m/second (24 m/minute), while the cut-off allowing unrestricted community walking is 0.8 m/second (48 m/minute) [2,10,19]. Schmid et al also reported that the mean walking speed of individuals capable of restricted community walking was 25.8-47.4 m/minute, compared to 48-72 m/minute for those capable of unrestricted community walking [10]; however, the above values refer to walking at a comfortable speed. An earlier report indicated that MWS is approximately 1.32 times higher than the comfortable walking speed, irrespective of walking speed [2]. Therefore, the MWS cut-offs for restricted and unrestricted community walking would be 31.7 and 63.4 m/minute, respectively. These values are close to those reported by a study from Japan, which found that the mean MWS for individuals restricted to indoor walking is 33.6 m/minute, compared to 61.8 m/minute for those capable of unassisted community walking [13]. Therefore, one of the inclusion criteria for secondary registration was MWS of 30-60 m/minute. We believe that one of the goals of HAL treatment is to improve the walking ability of patients with community walking and to restore them to society. Furthermore, we included the 6-minute walking distance as a secondary outcome, because this parameter was recently reported to be more suitable than MWS for evaluating walking ability and ADL function [20].

The duration and number of interventions are key factors affecting the effectiveness of the device. A review [21] of clinical research on conventional robot-assisted training revealed that many studies included at least 20 training sessions conducted over 4 weeks; of the studies that showed no effectiveness of robot-assisted intervention (versus a control group), two-thirds employed only 10-12 sessions. To date, the best training duration and frequency of robot-assisted training sessions remains unclear. A before-after study by Kawamoto et al [8] revealed that 16 sessions produced an improvement in walking speed. Meanwhile, the study by Watanabe et al [9] employed a 4-week program with 3 sessions per week and 20 minutes per session, for a total of 12 sessions, reporting limited effectiveness (only in terms of improving the functional ambulation category). Taken together, these earlier observations suggest an impact of reducing the length of training or the number of training sessions. Indeed, a pilot study [11] involving 5 sessions per week for 5 weeks, for a total of 25 sessions, reported an effective improvement in walking speed. Therefore, the present study uses an intervention protocol like that employed in the pilot study [11].

The present study has several limitations. Factors that affect the prognosis of stroke patients with hemiparesis include the extent of sensory impairment, the patient’s motivation, assistance from family members, and the structure of the rehabilitation plan. Another limitation is the inability to blind patients or investigators to the intervention after group allocation. The nature of rehabilitation research precludes the implementation of sham interventions, and it is thus impossible to eliminate bias caused by the placebo effect.

## Appendix

Multimedia Appendix 1 Assessment schedule.

## Abbreviations

ADL: activities of daily living
HAL: Hybrid Assistive Limb
MWS: maximum walking speed
PT: physical therapist
