# Supplementary material for: Effectiveness of a Walking Program Involving the Hybrid Assistive Limb Robotic Exoskeleton Suit for Improving Walking Ability in Stroke Patients: Protocol for a Randomized Controlled Trial
Source: JMIR Res Protoc. 2019 Oct 11;8(10):e14001. doi: 10.2196/14001 (PMC6913690; doi:10.2196/14001)
Supplement: Multimedia Appendix 1 [file resprot_v8i10e14001_app1.pdf]

Table 1. Assessment schedule.

Assessment	Baseline	Preintervention observation (weeks)													Intervention (weeks)						Postintervention observation (weeks)	
		1	2	3	4	5	6	・・[a]	X–5	X–4	X–3	X–2	X–1	X[b]	Baseline	1	2	3	4	5	1	2
Maximum walking speed (MWS) on the 10-m walk test	✓	✓	✓	✓	✓	✓	✓	✓	✓	✓	✓	✓	✓	✓	✓	✓	✓	✓	✓	✓	✓	✓
Mean step length and average step rate at MWS															✓	✓	✓	✓	✓	✓	✓	✓
Single-leg support time expressed as a percentage of the gait cycle at MWS															✓					✓		✓
Maximum distance walked within the span of 6 min (6-min walk test)															✓					✓		✓
Functional ambulation category															✓	✓	✓	✓	✓	✓	✓	✓
Berg Balance Scale score															✓					✓		✓
Leg score on Fugl-Meyer Assessment															✓					✓		✓

[a]・・：shows the transition of time.

[b]X: represents the day when the recovery of MWS is determined to be stagnant. Therefore, X-1 is the day before the day determined to be stagnant, and so on for X-2, X-3, etc.
